# Characterization of whole genome amplified (WGA) DNA for use in genotyping assay development

**DOI:** 10.1186/1471-2164-13-217

**Published:** 2012-06-01

**Authors:** Tao Han, Ching-Wei Chang, Joshua C Kwekel, Ying Chen, Yun Ge, Francisco Martinez-Murillo, Donna Roscoe, Živana Težak, Reena Philip, Karen Bijwaard, James C Fuscoe

**Affiliations:** 1 Division of Systems Biology, National Center for Toxicological Research, FDA, Jefferson, AR, 72079, USA; 2 Division of Personalized Nutrition and Medicine, National Center for Toxicological Research, FDA, Jefferson, AR, 72079, USA; 3 Division of Genetic and Molecular Toxicology, National Center for Toxicological Research, FDA, Jefferson, AR, 72079, USA; 4 Office of In Vitro Diagnostic Device Evaluation & Safety, Center for Devices and Radiological Health, FDA, Silver Spring, MD, 20993, USA

**Keywords:** Whole genome amplification, Array-based comparative genomic hybridization, TaqMan copy number assay, DNA sequencing

## Abstract

**Background:**

Genotyping assays often require substantial amounts of DNA. To overcome the problem of limiting amounts of available DNA, Whole Genome Amplification (WGA) methods have been developed. The multiple displacement amplification (MDA) method using Φ29 polymerase has become the preferred choice due to its high processivity and low error rate. However, the uniformity and fidelity of the amplification process across the genome has not been extensively characterized.

**Results:**

To assess amplification uniformity, we used array-based comparative genomic hybridization (aCGH) to evaluate DNA copy number variations (CNVs) in DNAs amplified by two MDA kits: GenomiPhi and REPLI-g. The Agilent Human CGH array containing nearly one million probes was used in this study together with DNAs from a normal subject and 2 cystic fibrosis (CF) patients. Each DNA sample was amplified 4 independent times and compared to its native unamplified DNA. Komogorov distances and Phi correlations showed a high consistency within each sample group. Less than 2% of the probes showed more than 2-fold CNV introduced by the amplification process. The two amplification kits, REPLI-g and GenomiPhi, generate very similar amplified DNA samples despite the differences between the unamplified and amplified DNA samples. The results from aCGH analysis indicated that there were no obvious CNVs in the CFTR gene region due to WGA when compared to unamplified DNA. This was confirmed by quantitative real-time PCR copy number assays at 10 locations within the CFTR gene. DNA sequencing analysis of a 2-kb region within the CFTR gene showed no mutations introduced by WGA.

**Conclusion:**

The relatively high uniformity and consistency of the WGA process, coupled with the low replication error rate, suggests that WGA DNA may be suitable for accurate genotyping. Regions of the genome that were consistently under-amplified were found to contain higher than average GC content. Because of the consistent differences between the WGA DNA and the native unamplified DNA, characterization of the genomic region of interest, as described here, will be necessary to ensure the reliability of genotyping results from WGA DNA.

## Background

Advances in genomic technologies have enabled development of many novel genome analysis methods that may have applications in the understanding, diagnosis, and management of genetic diseases and cancer. Comprehensive high-throughput assays are available for detection of single nucleotide polymorphisms (SNPs) [1-3], DNA copy number variation (CNV) [4], microsatellite expansion or contraction [5] and loss of heterozygosity (LOH) [6], all of which detect sometimes subtle genomic alterations associated with disease. Some of these assays require micrograms of DNA which may be difficult to obtain for many clinical samples. In addition, inadequate DNA template may prevent the performance of multiple assays on a single sample [7]. Limited availability of DNA also poses challenges for manufacturers and regulators of genetic diagnostic devices. One of these challenges is availability of sufficient quantities of DNA samples to appropriately validate a particular test under investigation, especially when uncommon mutations for a rare disease severely limit access to patient sample specimens.

Since the U.S. Food and Drug Administration (FDA)’s clearance of the first genotyping assay for cystic fibrosis (CF) in 2005, the Office of *In Vitro* Diagnostic Device Evaluation and Safety within the Center for Devices and Radiological Health (CDRH) has reviewed a number of other genotyping assays for inherited disorders. Some of these genotyping assays are intended to detect relatively rare heritable diseases consisting of multiple disease-causing alleles (mutations) for each disease, while others may detect more common diseases, but are intended to test very rare as well as common mutations. To show the accuracy of these assays, manufacturers would generally use patient samples (whole blood or archived DNA). For rare mutations or alleles, it is often difficult to obtain sufficient quantities of such clinical samples to adequately assess test performance. Therefore, there is an interest in using whole genome amplified (WGA) DNA samples created from patient samples instead of native DNA samples to increase the availability of appropriate samples to query the performance of the assays. WGA is a method that amplifies small amounts of genomic DNA several thousand-fold *in vitro*. The WGA process has the potential, however, to result in non-uniform amplification of the DNA in which some regions of the genome are over-represented and others are under-represented. Such biased amplification could make the WGA DNA unsuitable for some of the studies designed to assess the clinical assay performance.

Several WGA methods based on the polymerase chain reaction (PCR) with Taq polymerase were initially developed [8-12]. These methods included the use of primers directed at highly repetitive sequences [11], ligation of linkers to fragmented DNA [12], degenerate oligonucleotide primed PCR [9], and primer extension preamplification [10]. All of these methods suffer from a relatively high level of mutations in the amplified DNA (error rate 3x10^-5^[13]) and highly non-uniform amplification due to the low fidelity and low processivity of the Taq polymerase, respectively. In 2002, Dean et al. [14] described the multiple displacement amplification (MDA) technique. This method of WGA takes advantage of the high processivity and low error rate of the Φ29 bacteriophage DNA polymerase. This polymerase has a 3’-5’ proof-reading activity and adds an average of 70,000 templated nucleotides to a primer [15], resulting in higher fidelity and less biased amplification than with the Taq polymerase methods. The WGA process using Φ29 polymerase is isothermal and uses random primers to target the entire genome. The polymerase has strong strand displacement activity so that exponential amplification occurs through a branching mechanism [14], resulting in a high yield of DNA. The MDA process has been recently reviewed and shown to be superior to other DNA amplification methods with regard to genotyping, genomic coverage, and amplification bias [16,17].

MDA-based WGA has been frequently used in DNA sample preparation for genotyping and sequencing in recent years [18-21]. High call rates (97.5%) and excellent concordance rates were achieved from WGA samples using high-density SNP arrays [19,20]. There was a very low error rate (1 SNP genotyping error per 1000 assays) when high quality DNA was used as template [21]. MDA-based WGA has also been used for analyses of single cells [22,23]. Jiang et al. successfully amplified DNA from single sperm at least 250 fold with a single round of MDA [24]. DNA amplified from single lymphocytes was used for multiple analyses of 20 different loci including the ΔF508 deletion in exon 10 and two intragenic microsatellite markers in the CF gene [25]. Short tandem repeats (STR) and Human Leucocyte Antigen typing were performed using DNA amplified by MDA from a single cell [26]. MDA-WGA has also been increasingly used in the field of forensic testing [27,28]. Even in the often degraded DNA samples, WGA showed the capability and potential to increase the quality and quantity of DNA from difficult samples in forensic casework [29]. Although MDA-based WGA has been successfully applied in many studies, there are several potential problems that may affect interpretation of results. These include the finding that large amounts of nonspecific DNA amplification can be generated during MDA, mostly due to primer-directed DNA synthesis [5,30], and the quantity [21,31] and quality [32] of input DNA into the MDA reaction can affect genotyping results.

Despite the advantage of the MDA-based WGA method, the uniformity of the amplification process across the genome has not been extensively characterized [3,4,33]. In 2004, Paez et al. [33] examined DNAs amplified using a commercially available MDA method, REPLI-g, using 10 k Affymetrix SNP arrays and direct sequencing of ~500,000 bp of DNA and showed near-complete genome representation, as well as low replication error rate. In 2006, Pinard et al. [34] assessed the bias of WGA methods on bacterial genomes using massively parallel sequencing and found statistically significant amplification bias, although the MDA methods produced the least bias. Arriola et al. (2007) [35], using a second commercially available MDA method, GenomiPhi, evaluated the WGA DNA with low density array comparative genomic hybridization (aCGH), a method with higher resolution (~ 200 kb in this case) than the traditional CGH method [36]. Copy number biases were found, with the extent dependent on the degree of amplification. While these studies and others [16,17] have consistently shown non-uniformity of amplification by the MDA methods (although less than with other WGA methods), the uniformity of amplification has not been examined with high resolution aCGH at a resolution of several kb. In addition, the consistency of the amplification process has not been evaluated in replicate amplifications of the same sample, an important parameter when such material may be used for validation of genetic diagnostic devices or tests.

In this study, we evaluated two commonly used methods of WGA for their ability to produce large quantities of uniformly amplified DNA with minimal introduced mutations. Biases during the amplification process were determined by measurements of DNA CNVs at nearly 1 million positions in the genome using aCGH analysis. Particular attention was given to a single gene, CFTR, which encodes cystic fibrosis transmembrane conductance regulator. Mutations in CFTR can cause cystic fibrosis, a rare disease manifested by thick, sticky mucus and salty sweat, which usually leads to lung transplant or early death [37]. In addition to aCGH, copy number variation in the CFTR gene was examined by a second method, quantitative real-time PCR. Finally, the introduction of mutations during the WGA process was examined by comparing the DNA sequence of the resulting amplified DNA to that of the unamplified DNA. The consistency of the WGA process was evaluated by examining four replicate amplified DNAs derived from three individuals, one healthy and two with cystic fibrosis.

## Results

### CNV introduced by the WGA process

**Figure 1:**
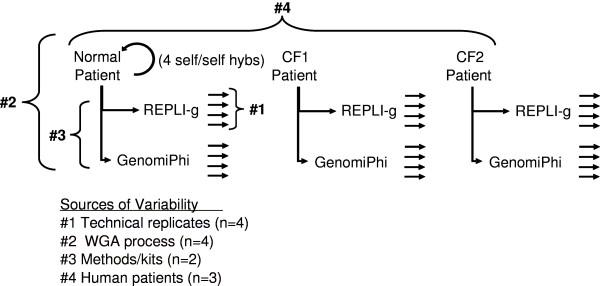
**aCGH experimental design with 4 replications each for GenomiPhi and REPLI-g**. DNAs used in this study were from one healthy individual (Normal) and two patients with cystic fibrosis (CF1 and CF2)

To characterize the consistency and faithfulness of whole genome amplification methods based on Ф29 polymerase, three human DNA samples (Table 1) were amplified using two commercially available kits, REPLI-g and GenomiPhi. The DNA samples were derived from a healthy individual (Normal) and two patients with cystic fibrosis (CF1 and CF2). The experimental design is shown in Figure 1. Each of the three DNA samples was amplified 4 independent times by both REPLI-g and GenomiPhi kits. DNA copy number changes introduced into the amplified DNA were evaluated by comparing the amplified DNA to the native unamplified DNA utilizing aCGH. The Agilent Human CGH array was used with the assumption that as a research tool it could comprehensively evaluate and reveal gross copy number changes at nearly 1 million loci. In addition, unamplified Normal samples were compared to unamplified Normal samples four times to define the technical variability of the aCGH process. Any copy number variation in such a self-self hybridization would be due to technical limits of the aCGH process since the DNA samples were identical. A total of 28 CGH arrays were used in this study.

**Table 1:** DNA sample information

**Sample**	**Visit/Blood**	**Ethnicity**	**Sex**	**Age**	**Genotype**	**Diagnosis**	**OD**_**260/280**_
	**Draw date**						
Normal	9/27/2002	Caucasian	M	52	Control		1.76
CF1	10/30/2003	Caucasian	M	26	2xF508	Cystic Fibrosis	1.84
CF2	10/30/2003	Caucasian	F	20	1xG542X	Cystic Fibrosis	1.83
					1x3659delC		

DNA samples were purchased from PrecisionMed, Inc. and dissolved in TE buffer (10 mM Tris, 1 mM EDTA at pH 8.0). There are different mutations in the two patients with cystic fibrosis (CF1 and CF2).

**Figure 2:**
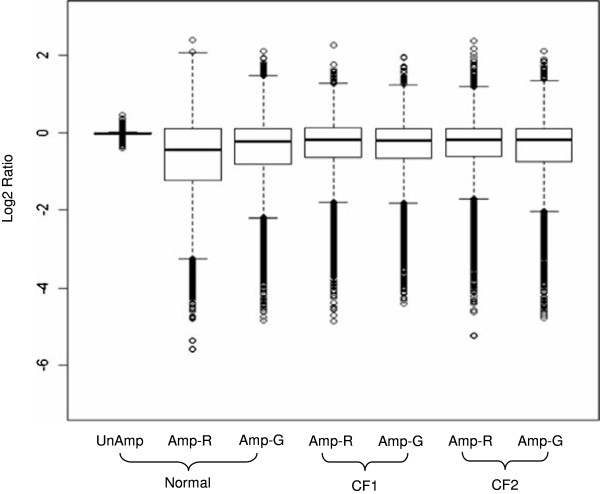
**Box plot of log2 ratios of samples to unamplified samples for Normal, CF1, and CF2 samples**. The average of the 4 replicates in each sample group is shown. UnAmp (no amplification); Amp-R (REPLI-g amplified); Amp-G (GenomiPhi amplified)

Figure 2 shows box plots of the average log_2_ ratios of amplified or unamplified test samples versus the corresponding native unamplified sample. The first plot shows the distribution of average log_2_ ratios of unamplified Normal DNA vs. unamplified Normal DNA (self-self hybridization). The distribution is centered at 0 and is very tight, indicating that little variability (i.e., CNV “noise”) is introduced by the aCGH process itself. Supporting this conclusion, the derivative log_2_ ratio spread (which is the average log ratio difference between adjacent probes of an array) for the 4 replicate self-self hybridizations was low (0.165). Thus, any change in the distribution of log_2_ ratios in amplified samples compared to unamplified samples could be considered to be due primarily to the WGA process. A notably wider distribution of the ratios was observed for the amplified samples compared to the unamplified samples although the median was still close to 0. These results suggest that CGH arrays can be sensitive tools for this type of CNV analysis. More importantly, WGA resulted in CNV changes for all three samples. The majority of the CNV changes were due to under-amplification.

To better understand differences in copy number introduced by the WGA process, we compared the log_2_ ratio distribution of the Normal unamplified DNA vs. Normal unamplified DNA (self-self) with the log_2_ ratio distribution of the WGA Normal (amplified) DNA vs. Normal unamplified DNA by calculating the Kolmogorov distances. The pair-wise comparisons for both the REPLI-g and GenomiPhi WGA methods are shown in Table 2. The median pair-wise Kolmogorov distances between the REPLI-g-amplified vs. unamplified distribution and GenomiPhi-amplified vs. unamplified distribution are 0.407 and 0.368, respectively, consistent with differences between the distributions of log_2_ ratios before and after WGA.

**Table 2:** Pair-wise comparison of Kolmogorov distances between probe ratio distributions of unamplified and amplified Normal patient DNA samples

			Sample		Kolmogorov distance			
		Normal UnAmp/UnAmp			
		Replicate 1			Replicate 2	Replicate 3	Replicate 4
NormalAmp-R/ UnAmp	Replicate 1	0.398	0.402	0.405	0.402
	Replicate 2	0.409	0.413	0.416	0.413
	Replicate 3	0.385	0.389	0.392	0.388
	Replicate 4	0.415	0.419	0.423	0.420
Normal Amp-G/UnAmp	Replicate 1	0.370	0.354	0.386	0.342
	Replicate 2	0.381	0.366	0.398	0.354
	Replicate 3	0.357	0.340	0.373	0.328
	Replicate 4	0.388	0.373	0.405	0.361

Each row represents probe ratio distributions from replications of amplified DNA samples; each column represents probe ratio distributions from replications of unamplified DNA samples. The median values are 0.407 and 0.368 for REPLI-g and GenomiPhi, respectively. UnAmp (unamplified samples); Amp-R (REPLI-g amplified samples); Amp-G (GenomiPhi amplified samples).

Another measure of the uniformity of the WGA process is the percentage of probes with ratios below or above a certain cut-off value when the WGA DNA is compared to the unamplified DNA. Table 3 shows the percentage of probes on the CGH arrays with ratios greater than 1.5-fold, 2-fold, 2.5-fold, 3-fold, and 4-fold for the three DNA samples and the two WGA methods. The percentages are the average of the 4 replicates. Also shown is the percentage of probes above these cut-offs for the unamplified Normal DNA compared to itself; all ratios were within 2, consistent with low variability of the aCGH method. Both amplification methods resulted in less than 1% of probes showing CNV of greater than 2-fold, with the exception of the Normal sample amplified by GenomiPhi (1.273%). If the fold-change criterion was relaxed to 3-fold, less than approximately 0.1% of probes showed CNV. Table 4 shows regions of the genome that were consistently under-amplified by at least 3-fold across all samples by both amplification methods. These under-amplified regions represent a small portion of total genome (2.1%), and contain about 1099 genes (approximately 4.4%). Thus, while the MDA technology resulted in changes in copy number, the changes were relatively small in most regions of the genome, and were concentrated in a relatively small number of chromosomal loci.

**Table 3:** Average CNV at various cut-off ratios

Sample	Comparison*	Percentage with ratios > 1.5	Percentage with ratios > 2.0	Percentage with ratios > 2.5	Percentage with ratios > 3.0	Percentage with ratios > 4.0
Normal	UnAmp/UnAmp	0.004	0.000	0.000	0.000	0.000
	Amp-R/UnAmp	2.043	0.525	0.194	0.077	0.010
	Amp-G/UnAmp	8.139	1.273	0.339	0.109	0.017
CF1	Amp-R/UnAmp	2.191	0.478	0.162	0.057	0.010
	Amp-G/UnAmp	3.682	0.747	0.230	0.074	0.015
CF2	Amp-R/UnAmp	1.913	0.486	0.192	0.075	0.007
	Amp-G/UnAmp	2.883	0.605	0.183	0.061	0.015

* Indicates samples on the CGH array. The averages of the 4 replicate WGA are given. UnAmp (unamplified samples); Amp-R (REPLI-g amplified samples); Amp-G (GenomiPhi amplified samples).

**Table 4:** Common highly under-amplified regions (>3 fold) in Normal, CF1, and CF2 samples after WGA

Chromosome	Cytoband	Stat (bp)	Stop (bp)	No. of Probes	No. of Genes*
Chr1	p36.33 - p36.32	787630	3693246	967	88
Chr2	q37.3	241165907	241864699	258	13
Chr4	p16.3	533101	3842102	668	38
Chr5	p15.33	25942	2285711	724	30
Chr7	p22.3	42776	2759847	734	30
Chr8	q24.3	142067269	146261757	1480	110
Chr9	q34.2 - q34.3	136872507	140734384	1500	106
Chr10	p15.3	499391	681067	86	1
Chr11	p15.5 - p15.4	271403	3216315	997	78
Chr12	p13.33-p13.31	163393	7069315	87	6
Chr13	q34	114305327	114924254	174	8
Chr14	q32.31-p32.32	101469473	105943028	741	63
Chr16	p13.3	93428	3286713	1256	169
Chr16	q24.2 - q24.3	87871999	89999459	555	35
Chr17	p13.3	47346	1964509	814	13
Chr17	q25.2-p25.3	74886218	80217558	601	62
Chr18	q23	77064970	77617915	219	6
Chr19	p13.3	259195	2442313	983	103
Chr20	q13.33	60782793	62819250	880	91
ChrX	q28	152713107	153194997	218	23
ChrY	p11.32	10891	1663423	443	19

Cytobands and chromosomal locations are based on hg 19 (NCBI Genome build 37).

* total number of genes located within the under-amplified regions.

### Consistency of the WGA process

Differences in the distribution of ratios within each sample group were calculated using the Kolmogorov distance. Table 5 shows pair-wise comparisons of Kolmogorov distances within each sample group, as well as the median value for each group. The median Kolmogorov value for the four self-self hybridizations (Normal sample, unamplified vs. unamplified) was 0.025 and the median Kolmogorov values within the other sample groups were similarly low. Thus, the WGA process appears to be reproducible in terms of uniformity of amplification throughout the genome. The Phi correlation was calculated to evaluate the consistency of the position of the CNV loci (defined as having a ratio of greater than 2). Higher values (greater than 0.7) indicate the ratios at each probe location are very similar. Results of pair-wise Phi correlations (Table 6) indicate that the majority of the replicates within each sample/amplification group are highly correlated (similar patterns of CNVs). The exception is the CF1 sample, which has a Phi correlation just below 0.7. The Phi correlation values are above 0.7 if the fold cut-off is set at 2.5 (data not shown). Overall, the results from the Kolmogorov distances and Phi correlations show that there is high consistency in the WGA process within each sample/WGA method group.

**Table 5:** Pair-wise comparison of Kolmogorov distances between probe ratio distributions within each sample group

			Sample*		Kolmogorov distance			
		Replicate 1			Replicate 2	Replicate 3	Replicate 4
Normal	Replicate 1	0		**0.025**	
UnAmp/UnAmp	Replicate 2	0.019	0		
	Replicate 3	0.026	0.044	0	
	Replicate 4	0.024	0.018	0.049	0
Normal	Replicate 1	0		**0.013**	
Amp-R/UnAmp	Replicate 2	0.009	0		
	Replicate 3	0.011	0.005	0	
	Replicate 4	0.015	0.020	0.021	0
Normal	Replicate 1	0		**0.029**	
Amp-G/UnAmp	Replicate 2	0.026	0		
	Replicate 3	0.020	0.033	0	
	Replicate 4	0.048	0.023	0.053	0
CF1	Replicate 1	0		**0.085**	
Amp-R/UnAmp	Replicate 2	0.108	0		
	Replicate 3	0.046	0.062	0	
	Replicate 4	0.065	0.062	0.120	0
CF1	Replicate 1	0		**0.053**	
Amp-G/UnAmp	Replicate 2	0.050	0		
	Replicate 3	0.056	0.023	0	
	Replicate 4	0.042	0.082	0.095	0
CF2	Replicate 1	0		**0.047**	
Amp-R/UnAmp	Replicate 2	0.003	0		
	Replicate 3	0.065	0.066	0	
	Replicate 4	0.029	0.029	0.088	0
CF2	Replicate 1	0		**0.039**	
Amp-G/UnAmp	Replicate 2	0.018	0		
	Replicate 3	0.050	0.036	0	
	Replicate 4	0.055	0.043	0.009	0

* Indicates samples on CGH array.

The median values for each group are shown in bold. UnAmp (unamplified samples); Amp-R (REPLI-g amplified samples); Amp-G (GenomiPhi amplified samples).

**Table 6:** Pair-wise comparison of Phi correlations of probe ratios greater than 2 within each sample group after CBS smoothing

			Sample*		Phi correlation value			
		Replicate 1			Replicate 2	Replicate 3	Replicate 4
Normal	Replicate 1	1		**1.000**	
UnAmp/UnAmp	Replicate 2	1.000	1		
	Replicate 3	1.000	1.000	1	
	Replicate 4	1.000	1.000	1.000	1.000
Normal	Replicate 1	1		**0.842**	
Amp-R/UnAmp	Replicate 2	0.855	1		
	Replicate 3	0.840	0.843	1	
	Replicate 4	0.877	0.840	0.809	1
Normal	Replicate 1	1		**0.805**	
Amp-G/UnAmp	Replicate 2	0.853	1		
	Replicate 3	0.839	0.796	1	
	Replicate 4	0.773	0.814	0.707	1
CF1	Replicate 1	1		**0.611**	
Amp-R/UnAmp	Replicate 2	0.597	1		
	Replicate 3	0.764	0.665	1	
	Replicate 4	0.457	0.625	0.520	1
CF1 Amp-G/UnAmp	Replicate 1	1		**0.667**	
	Replicate 2	0.687	1		
	Replicate 3	0.679	0.777	1	
	Replicate 4	0.632	0.656	0.589	1
CF2	Replicate 1	1		**0.768**	
Amp-R/UnAmp	Replicate 2	0.824	1		
	Replicate 3	0.697	0.744	1	
	Replicate 4	0.796	0.793	0.707	1
CF2	Replicate 1	1		**0.780**	
Amp-G/UnAmp	Replicate 2	0.865	1		
	Replicate 3	0.798	0.746	1	
	Replicate 4	0.762	0.728	0.838	1

* Indicates samples on CGH array.

The median Phi correlation values from top to bottom are shown in bold. UnAmp (unamplified samples); Amp-R (REPLI-g amplified samples); Amp-G (GenomiPhi amplified samples).

### Comparisons between the two whole genome amplification kits

Both WGA kits used in this study, REPLI-g and GenomiPhi, use the same bacterial phage Φ29 DNA polymerase. However, the assay procedures are considerably different with regard to reaction time (4 hr with GenomiPhi and 10 hr with REPLI-g) and method of DNA denaturation (heat with GenomiPhi and alkaline with REPLI-g). Thus, comparison of outcomes of these procedures is important. The median values of pair-wise Kolmogorov distances for Normal, CF1, and CF2 samples between REPLI-g and GenomiPhi amplified samples are 0.046, 0.055, and 0.046, respectively (Table 7) which are similar to the Kolmogorov distances from samples amplified by the same method (range: 0.013- 0.085; see Table 4). The median pair-wise Phi correlations for Normal, CF1, and CF2 samples between REPLI-g and GenomiPhi amplified samples after CBS smoothing are 0.739, 0.789 and 0.792, respectively (Table 8). Again, these values are similar to those obtained when comparing within an amplification method (see Table 6). These results indicate that the two amplification methods generate similar amplified DNA samples despite the procedural differences.

**Table 7:** Pair-wise comparison of Kolmogorov distances of distribution ratios between the REPLI-g and GenomiPhi methods

			Sample		Kolmogorov distance			
		Amp-G/UnAmp			
		Replicate 1			Replicate 2	Replicate 3	Replicate 4
Normal	Replicate 1	0.031	0.055	0.026	0.078
Amp-R/UnAmp	Replicate 2	0.038	0.063	0.035	0.085
	Replicate 3	0.041	0.065	0.037	0.088
	Replicate 4	0.032	0.051	0.019	0.072
CF1	Replicate 1	0.095	0.050	0.041	0.131
Amp-R/UnAmp	Replicate 2	0.017	0.061	0.071	0.025
	Replicate 3	0.050	0.009	0.019	0.085
	Replicate 4	0.079	0.115	0.130	0.037
CF2	Replicate 1	0.082	0.069	0.033	0.027
Amp-R/UnAmp	Replicate 2	0.083	0.069	0.034	0.028
	Replicate 3	0.021	0.019	0.034	0.039
	Replicate 4	0.106	0.095	0.060	0.052

The median values for Normal, CF1, and CF2 groups between the WGA methods are 0.046, 0.055, and 0.046, respectively. Amp-R (REPLI-g amplified); Amp-G (GenomiPhi amplified).

**Table 8:** Pair-wise comparisons of Phi correlations of probe ratios greater than 2 between GenomiPhi and REPLI-g amplified samples

			Sample		Phi correlation value			
		Amp-G/UnAmp			
		Replicate 1			Replicate 2	Replicate 3	Replicate 4
Normal	Replicate 1	0.746	0.724	0.782	0.725
Amp-R/UnAmp	Replicate 2	0.729	0.712	0.783	0.706
	Replicate 3	0.688	0.664	0.751	0.653
	Replicate 4	0.814	0.772	0.811	0.763
CF1	Replicate 1	0.736	0.804	0.906	0.681
Amp-R/UnAmp	Replicate 2	0.842	0.782	0.711	0.807
	Replicate 3	0.828	0.804	0.815	0.738
	Replicate 4	0.733	0.692	0.622	0.796
CF2	Replicate 1	0.762	0.771	0.833	0.835
Amp-R/UnAmp	Replicate 2	0.776	0.773	0.831	0.818
	Replicate 3	0.789	0.839	0.782	0.795
	Replicate 4	0.736	0.741	0.825	0.828

The median values for Normal, CF1, and CF2 groups were 0.738, 0.789, and 0.792, respectively.

**Figure 3:**
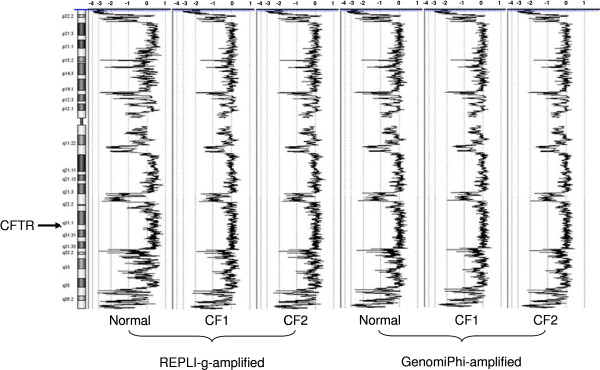
**Patterns of CNVs on chromosome 7 with amplified samples after smoothing (CBS). The average of the 4 replicates in each sample group is shown and compared with the unamplified sample DNA**. The CFTR gene is located at cytoband 7q31.2 on chromosome 7. The numbers along the top of the graphs indicate log 2 ratios of probes

Visual comparison of CNV generated by these amplification methods across chromosome 7 is illustrated in Figure 3. The pattern of CNVs is similar between the 2 amplification methods and among the 3 DNA specimens, with much of the chromosome showing CNV < 2-fold when compared to the unamplified DNA. However, regions with consistent >2-fold CNV are evident near the chromosomal ends, as well as near 7q21.3, 7q11.22, and 7p14.1, among others. The region near the CFTR gene appears to amplify uniformly and contains no CNVs greater than 2-fold.

### Quantitative real-time PCR copy number assay within the CFTR gene region

**Figure 4:**
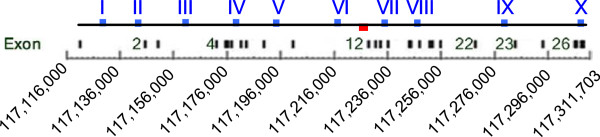
**A schematic diagram of the PCR probe locations and a 2-kb sequencing region of the CFTR gene on chromosome 7.** The blue boxes show the locations of the PCR probes (see Table 8). The small red box highlights the 2 kb region of the CFTR gene that was sequenced. This DNA segment includes part of intron 11 and exon 12, which account for 1% of the gene

**Figure 5:**
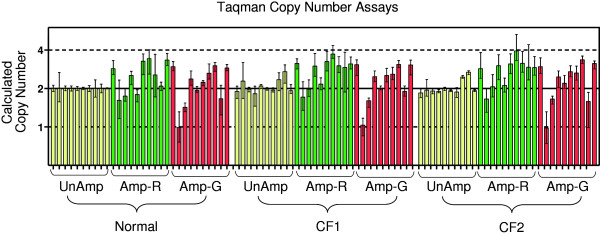
**Quantitative real time PCR copy number assays**. Taqman assays were used to examine copy number variations at 10 locations within the CFTR gene in both the unamplified and amplified DNA samples. Average calculated copy number values are plotted with bars representing minimum and maximum values from replicate measurements (n = 4). Within each amplification / sample group, the order of the probe results is I- X (see Figure 4). UnAmp (no amplification); Amp-R (REPLI-g amplified); Amp-G (GenomiPhi amplified)

Subsequent to array analysis, a quantitative PCR method was used to further evaluate the variability between the amplified and unamplified DNA samples. Ten TaqMan Copy Number PCR probes were selected from Applied Biosystem’s pre-designed research assays across the length of the CFTR gene, a well-studied genetic disease locus, representing 9 introns and 1 exon (Table 9 and Figure 4). Relative copy number was calculated for each probe in reference to the unamplified DNA sample for each subject. Each of the ten probes gave comparable results showing less than 2-fold difference (considering both over- and under-amplification) in fold-change between amplified and unamplified DNA samples (Figure 5). This low level of variability was also evident when comparing results between the two amplification methods (REPLI-g and GenomiPhi), suggesting comparable results irrespective of the manufacturer’s protocol. Furthermore, the amount of variability across the three human samples was similar, suggesting robust amplification results regardless of possible biological differences. Thus, the TaqMan Copy Number PCR results are in agreement with the aCGH data (Figure 3) in identifying low levels (below 2-fold change) of amplification variability introduced by the WGA process in the CFTR locus.

**Table 9:** Probe information for TaqMan Copy Number Quantitative PCR

PCR Probe	Applied Biosystems probe ID	CFTR location	Chromosome location (Approx.)
I	Hs05020079_cn	Intron 1	Chr7: 117122192
II	Hs04952703_cn	Intron 2	Chr7: 117145581
III	Hs04988506_cn	Intron 3	Chr7: 117153814
IV	Hs04984230_cn	Intron 9	Chr7: 117185643
V	Hs04963787_cn	Intron 11	Chr7: 117203673
VI	Hs05017940_cn	Intron 12	Chr7: 117228242
VII	Hs05001680_cn	Intron 15	Chr7: 117238610
VIII	Hs04963453_cn	Intron 18	Chr7: 117247359
IX	Hs04947556_cn	Intron 23	Chr7: 117282984
X	Hs00393982_cn	Intron 27	Chr7: 117307221

Chromosome locations are based on NCBI Human Genome Build 37.

### DNA sequence analysis in the CFTR gene region

Bidirectional Sanger DNA sequencing was performed on an approximately 2 kb region of the CFTR gene to examine the fidelity of the WGA process using primers shown in Table 10 (see Methods). DNA sequences from the WGA samples were compared with the respective unamplified DNA samples. The results are shown in Table 11 and there were no detectable mutations introduced by either WGA method in the 3 DNA samples (approximate 7,900 nucleotides examined for each sample/WGA method). Thus, the level of detectable mutations introduced by the GenomiPhi and REPLI-g WGA methods in this analysis was less than 1 mutation per 23,000 nucleotides examined. These results suggest that both GenomiPhi and REPLI-g appear to be robust and accurate methods for amplifying nanogram quantities of starting DNAs to microgram levels.

**Table 10:** Sequencing primer information

Primer Name	Primer location	Primer sequence (5’- 3’)	Tm	Amplicon size (bp)
Seq1_F	112776-112797	GGACATCTCCAAGTTTGCAGAG	66	760
Seq1_R	113535-113512	GAAACATTTGACATCAGAGTCAC	64	
Seq2_F	113459-113479	GTCAAGGAGAGAGCTTTGTGG	64	729
Seq2_R	114187-114165	TGGACAACACATTACACATTCTG	64	
Seq3_F	114050-114071	GGCTTCTAGACATCCAACATAG	64	756
Seq3_R	114805-114784	GATAGCAGTGCTGCCACAACTG	68	

Primer locations (chromsome 7) are based on NCBI reference sequence NG_016465.1. “F” –forward primer; “R”- Reverse primer.

**Table 11:** Mutations in the WGA DNA

Sample	Amplification method	Nucleotides examined	Mutation	
			Total number	Percentage
Normal	REPLI-g	7900	0	<0.01
CF1	REPLI-g	7924	0	<0.01
CF2	REPLI-g	7909	0	<0.01
Normal	GenomiPhi	7888	0	<0.01
CF1	GenomiPhi	7905	0	<0.01
CF2	GenomiPhi	7906	0	<0.01

Nucleotides examined are the sum of 2-kb sequences from the four replications.

## Discussion

The sequencing of the human genome, coupled with advances in genomics technologies, is having a major impact on understanding basic human biology, as well as the molecular causes of diseases and toxicities [38]. In fact, the leaders of the U.S. Food and Drug Administration (FDA) and the National Institutes of Health (NIH) have recently outlined the opportunities in clinical medicine that are being created by advances in basic science, including genomics [39]. The two agencies have announced a new collaborative effort to focus regulatory and translational sciences on bringing medical products and therapies into the age of personalized medicine. This growing store of knowledge has enormous potential application to the development of devices and tests for use in the diagnosis, mitigation, treatment, cure, and prevention of disease and other conditions.

As of 2010, more than 100,000 germline mutations in more than 3700 genes have been associated with human inherited disease, with about 300 new disease genes and 10,000 mutations being identified annually [40]. In principle, DNA tests could be developed for each of these conditions. Development and approval of such tests for clinical diagnostics often require that accuracy be demonstrated on patient samples. That is, the test needs to detect the disease-causing mutation with high precision and accuracy. In addition, measures of proficiency are required by testing laboratories to ensure continued accuracy of the results. The Clinical Laboratory Improvement Advisory Committee has recently issued a good practices report for molecular genetic testing and has stressed the need for performance assessments [41]. Such recommendations are supportive of the 2008 Report of the Secretary’s Advisory Committee on Genetics, Health, and Society (SACGHS) [42]. Additionally, the SACGHS has identified the development of genetic reference materials as one of five critical gaps in the oversight system of genetic testing [42]. The device approval process, use of reference and control materials, and ongoing proficiency assessments require relatively large quantities of clinical samples in order to assure test performance is adequate and maintained over time. The availability of sufficient samples with appropriate mutations has been recognized as a critical issue in genetic testing, given the paucity of validated clinical inherited disease gene samples [43]. Therefore, methods that can expand the limited supply of validated clinical samples would have major impacts on both the test and device performance evaluation and approval process, and the on-going proficiency assessment of the test providers.

The importance of the amount of template DNA for MDA-based WGA has been thoroughly discussed for SNP assays and STR genotyping in previous studies [28,31]. For example, large number of SNPs could be accurately detected from as low as 0.01 ng of DNA template [27,28] and even degraded DNA samples can be used for forensic SNP typing [29], while over 100 ng of DNA template was needed for optimal STR genotyping [31]. In this study, we mainly focused on evaluating the uniformity and fidelity of WGA DNAs using CGH arrays, TaqMan copy number assays, and DNA sequencing. The results indicated the amplified DNA and its native unamplified DNA that we examined were similar, although not identical, in terms of DNA copy number variation. Importantly, no detectable introduced mutations were found under our experimental conditions.

The Agilent Human CGH array used in this study includes approximately 970,000 probes at 1 to 2 kb intervals throughout the human genome. To ensure the quality of this study, four independent replications for both the WGA and aCGH processes were used. The self-self hybridization of unamplified Normal DNA samples provided us the base-line measurement of noise for aCGH technology. The box plot of unamplified Normal samples indicated less than 0.004% of the approximately 970,000 probes on the CGH array have fold changes greater than 1.5 and no CNVs can be detected by aCGH (Figure 2 and Table 3). The low Kolmogorov distances (0.025) and high Phi correlations (1.00) between the four replicates of unamplified samples further showed the aCGH technology was reproducible and could be a reliable tool to access variability of the WGA process.

The WGA technologies have evolved over the years from Taq DNA polymerase-based (PCR-based) to bacteriophage Ф29 DNA polymerase-based methods (MDA). We focused on MDA methods in this study because of the processivity and reportedly low replication error rate of the Ф29 DNA polymerase. In this study, we compared two MDA-based commercial WGA kits: the REPLI-g and GenomiPhi kits. The comparison between the unamplified and amplified DNA samples showed that relatively few probes (approximately 1% or less) were over- or under-amplified by more than 2-fold (Table 3). GenomiPhi-amplified DNA, however, showed consistently more CNVs than the REPLI-g-amplified DNA. Kolmogorov distances also indicated that differences in the distribution of DNA copy number existed between the unamplified and amplified samples (Table 2), and that these differences were greater than the differences between the REPLI-g and GenomiPhi amplified samples (Table 7). Thus, even though the kits differ in method of DNA denaturation (heat vs. alkaline), buffer composition, and reaction time (4 hr vs. 10 hr), they produce similar amplified DNA, likely the result of the common Ф29 DNA polymerase.

The data in this report indicate that the two WGA methods examined can consistently amplify small amounts of DNA (ng) to large quantities (~40 μg) with relatively small changes in DNA copy number along the chromosomes. Changes in DNA copy number of greater than 3-fold are evident as under-amplified regions at the ends of chromosomes, as illustrated in Figure 3, and in discrete regions on many of the chromosomes (Table 4). Because of the consistency of the DNA amplification, knowledge of the positions of likely under-amplification can prevent the inappropriate use of WGA DNA. Results presented in Table 4 may serve as a reference guide to avoid target genes in these regions. Inspection of the GC content within these consistently under-amplified regions showed an average GC content of nearly 53% which was higher than the overall GC content of the human genome of 41%. Further investigations will be needed to evaluate the significance of this observation.

Cystic fibrosis (CF) is caused by severe dysfunction of cystic fibrosis transmembrane conductance regulator (CFTR), which commonly leads to progressive lung disease and a shortened life [44]. Currently, there is no cure available for CF, even though multiple interventions have been developed to slow its progression. Since the FDA cleared the first genotyping assay for CF in 2005, manufacturers have developed a number of genotyping tests for rare heritable diseases caused by multiple mutant alleles, using patient samples to assess performance. There is an increased interest in using WGA DNA samples created from patient samples to support the performance of the assays. This study shows that within certain limits, the WGA process produces large quantities of DNA that may be useful for this purpose. To address the concerns of mutations introduced by WGA, TaqMan copy number assays were used to analyze CNVs at 10 locations within CFTR gene region and a random selected 2 kb region in CFTR gene was also sequenced. Results from the TaqMan copy number assay are in excellent agreement with the aCGH results. The DNA sequencing analysis showed there were no mutations induced by WGA in this approximately 2 kb region of the CFTR gene (mutation induction was less than 4 x 10^-5^), which indicates that WGA DNAs can be used for enrichment of DNA samples for cystic fibrosis genotyping assays.

The objective of the study presented here was to determine whether WGA amplified samples may be a reliable alternative to native clinical specimens for assessing the performance of a test under investigation. The conclusions from this study provide scientific input that may serve to support regulatory decisions in the ascertainment of safety and effectiveness of diagnostic products that use whole genome amplified samples in clinical studies. This study may serve as a guide to the technical qualification of WGA DNA for assessing the performance of genotyping assays.

## Conclusions

In summary, WGA generates large quantities of DNA with relatively high uniformity and low replication error rate when compared to unamplified DNA. This suggests that WGA DNA may be suitable for accurate genotyping. However, because there are consistent differences between the WGA DNA and the native unamplified DNA, characterization of the genomic region of interest, as described here, will be necessary to ensure the reliability of genotyping results from WGA DNA.

## Methods

DNA Samples: DNA samples used in this study were purchased from PrecisionMed, Inc. (San Diego, CA) and are shown in Table 1. All three DNA samples were extracted from human blood and were dissolved in TE buffer (1 mM EDTA, 10 mM Tris at pH 8.0). The concentration and purity (A260/280 ratio) of chromosomal DNA was measured by the NanoDrop 1000 and PicoGreen methods (Life Technologies, Carlsbad, CA). DNA samples were aliquoted to four batches and stored at −20 °C before use.

Whole Genome Amplification (WGA): Two WGA kits, GenomiPhi (GE Healthcare, Piscataway, NJ ) and REPLI-g (Qiagen, Inc., Hilden, Germany), were used in this study. 20 ng of DNA template was used for the amplification process using the manufacturers' recommended protocols (Illustra GenomiPhi HY DNA Amplification kit protocol and REPLI-g Mini/Midi Handbook). The GenomiPhi reaction was allowed to proceed for 4 hr while the REPLI-g reaction was allowed to proceed for 10 hr. Both kits use the same phage Φ29 DNA polymerase to amplify the DNA templates, although the buffer components are proprietary. The average yields after WGA with GenomiPhi and REPLI-g were 37.5 μg and 14.0 μg DNA, respectively.

Array Comparative Genome Hybridization (aCGH): The Agilent (Santa Clara, CA) 1 M human CGH array used in this study is based on NCBI Build 37 (UCSC) with 963,029 biological features and 6,685 controls. The majority of the probes on this array have 1 to 2 kb spacing along the human chromosomes. DNA labeling and hybridization were performed following the Agilent Oligonucleotide Array-Based CGH for Genomic DNA Analysis protocol (V 6.1, 2009). One μg of genomic DNAs were labeled with fluorescent dyes (Cy3 or Cy5-dUTP). In this study, the amplified DNA samples were labeled with Cy3 and unamplified DNA samples were labeled with Cy5. Labeled DNAs with specific activity greater than 20 pmol of dye/μg DNA were used for aCGH. Labeled amplified (Cy3) and unamplified (Cy5) DNA samples were paired and co-hybridized to the arrays at 65 °C for 40 hrs, then washed at room temperature following the Agilent Oligonucleotide Array-Based CGH for Genomic DNA Analysis protocol (V 6.1, 2009). The hybridized array was immediately scanned with an Agilent DNA Microarray Scanner (Agilent Technologies, Inc.) at 2 μm resolution. The resulting images were analyzed by quantifying the Cy3 and Cy5 fluorescence intensity at each feature on the array using the Agilent Feature Extraction Software (V10.5). The fluorescence intensity of each pixel within the feature was determined and the median fluorescence of these pixel measurements was taken as the measure of fluorescence for the whole feature after subtraction of background. Dye bias was removed by linear normalization using the Agilent Feature Extraction Software before the intensity values were used to calculate ratios at each feature.

aCGH Data Analysis: Copy number variation (CNV) was calculated at each locus along each chromosome as the ratio of the sample (either unamplified or amplified) to the unamplified sample intensities. A modified algorithm [45] of the circular binary segmentation smoothing method (CBS, [46]) was used to partition these ratio measurements into chromosomal regions containing loci with equal copy numbers. Regions with ratios less than 2 were then defined as being unchanged (no CNV). Two approaches were used to measure the similarity of CNV patterns. To measure the difference between the distributions of ratios from different experimental groups, Kolmogorov distances were calculated which are the sub-distances between the distribution functions [47]. Low values indicate little difference between distributions. Phi correlation, which is a measure of association for two binary variables [48], was calculated to measure the consistency of locations of the CNVs. A low Kolmogorov distance coupled with a high Phi correlation indicates highly uniform amplification. Agilent Genomic Workbench 6.0 software (Agilent Technologies, Inc.) was also used to analyze CNVs along the chromosomes utilizing the CBS module. Raw data were imported into Agilent Genomic Workbench 6.0 for CNV analysis. The four replicates for each sample were combined based on the weight of each sample which is proportional to its quality in Agilent Genomic Workbench before analysis. Average aberration reports (minimal 3 consecutive probes within each section) for Normal, CF1, and CF2 after WGA were generated using the CBS method with a fold change cut-off at 3-fold. Cytobands with aberrations greater than 3 fold across the six amplified samples are summarized in Table 4.

Quantitative Real Time PCR Copy Number Assays: Unamplified and amplified genomic DNAs (10 ng) were used as templates in TaqMan Copy Number Assays (Applied Biosystems, Carlsbad, CA) according to the manufacturer’s protocol. Ten probes, distributed across the length of the CFTR gene as illustrated in Figure 4, were used to quantify copy number in each DNA sample using RNase P as the reference assay. The Applied Biosystems probe IDs and locations in the CFTR gene can be found in Table 9. Cycle threshold (Ct) values were exported into Applied Biosystems CopyCaller Software to calculate the copy number at each locus. Copy number values were standardized to that of the unamplified DNA sample, according to software recommendation. Average relative copy number values were plotted along with minimum and maximum values of replicate measurements (n = 4).

DNA Sequencing Analysis: Unamplified and WGA DNAs were sent to SeqWright, Inc. (Houston, TX) for determination of the DNA sequence of a 2 kb region within the CFTR gene. The 2 kb region was PCR- amplified as 3 overlapping segments of 760 bp, 729 bp, and 756 bp as shown in Table 10 and Figure 4. The DNAs for sequencing included the 4 replicate WGA samples for each WGA method/DNA sample group (24 samples) plus one sample of each of the unamplified DNA samples. The DNA sequence of these amplicons was determined by bidirectional fluorescent dye-terminator chemistry using an ABI Prism 3730xl DNA sequencer. The sequencing data were aligned using Sequencher software (Gene Code Corporation, Ann Arbor, MI), which is based on an optimized Smith-Waterman algorithm. Nucleotide positions in which no base call was made or in which the forward base call was different from the reverse base call were excluded from further analysis. Of 23,744 bases examined after each amplification method, 53 bases (0.2%) and 11 bases (0.05%) were excluded after amplification with GenomiPhi and REPLI-g, respectively.

## Competing interests

The authors declare that they have no competing interests.

## Authors’ contributions

JCF, TH, FMM, DR, ZT, RP, and KB devised the project. TH and JCF were responsible for experimental design, data interpretation and writing the manuscript. CWC performed the statistical analysis. TH performed the aCGH analysis. JCK did the TaqMan copy number assay. YC and YG did sequencing alignment analysis. All authors read and approved the final manuscript.
